# Feasibility of replacing fish oil with sunflower oil on the growth, body composition, fatty acid profile, antioxidant activity, stress response, and blood biomarkers of *Labeo rohita*

**DOI:** 10.1371/journal.pone.0299195

**Published:** 2024-03-14

**Authors:** Muhammad Asghar, Noor Khan, Mahroze Fatima, Murat Arslan, Simon John Davies, Naveed ul Haque

**Affiliations:** 1 Department of Fisheries and Aquaculture, University of Veterinary and Animal Sciences, Lahore, Pakistan; 2 Department of Aquaculture, Faculty of Fisheries, Ataturk University, Erzurum, Turkey; 3 Aquaculture Nutrition Research Unit, ANRU, Carna Research Station, School of Natural Sciences and Ryan Institute, University of Galway, Carna, Co., Galway, Ireland; 4 Department of Animal Nutrition, University of Veterinary and Animal Sciences, Lahore, Pakistan; Tamil Nadu Dr J Jayalalithaa Fisheries University, INDIA

## Abstract

A 90-day study was conducted to investigate the effects of substituting sunflower oil (SFO) for fish oil (FO) on various parameters in *Labeo rohita* (initial weight 18.21 ± 0.22 g). Five experimental diets with different levels of SFO (up to 7%) substitution for FO (0%, 25%, 50%, 75%, and 100%) were formulated, ensuring equal levels of nitrogen and lipids. The results indicated that even with 100% substitution of SFO with FO, there were no significant differences (P>0.05) were observed in growth performance. The survival rate (SR), hepato-somatic index (HSI), and viscero-somatic index (VSI) as well as whole-body composition were also nonsignificant by SFO substitution. However, the fatty acid profiles in both muscle and liver were influenced (P<0.05) by dietary substitution. Saturated fats (SFA) decreased, while monounsaturated fats (MUFA), and linoleic acid (LA) increased (P<0.05). On the other hand, the contribution of linolenic acid (ALA), docosahexaenoic acid (DHA), and eicosapentaenoic acid (EPA) decreased (P<0.05) as the amount of SFO in the diet increased. Hematology parameters, including red blood cells (RBCs), hemoglobin (Hb), and hematocrit (Hct), were not affected. Globulin (GLO) levels decreased significantly (P<0.05), while alanine transaminase (ALT) and aspartate transaminase (AST) activity showed nonsignificant increases (P>0.05). Total protein (TP) increased (P<0.05) at 100% SFO inclusion in the diet, and albumin (ALB) levels increased (P<0.05) at 75% and 100% SFO inclusion in the diet. Cholesterol (CHOL), triacylglycerol (TG), and high-density lipids (HDL) were not significantly affected (P>0.05), while low-density lipids (LDL) were significantly increased (P<0.05) compared to the control group. Cortisol (CORT) and glucose (GLU) levels showed nonsignificant (P>0.05) changes. Superoxide dismutase (SOD), glutathione peroxidase (GSH-Px) and catalase (CAT) activities in the liver and serum were not significantly (P>0.05) affected, while malondialdehyde (MDA) status was significantly (P<0.05) reduced. In conclusion, the fatty acid profile of the muscle and liver of fish was modified by the diets, and FO can be substituted with SFO up to 100% for *L*. *rohita*, which is beneficial for growth and immunity while marinating the lipid contents in fish. Our study revealed that fully replacing fish oil with SFO shows promise in fully replacing FO without compromising the growth and overall health status of the fish.

## Introduction

In aquaculture, fish oil (FO) has traditionally been a crucial source of dietary lipids due to its high content of beneficial long-chain polyunsaturated fatty acids (LC-PUFAs), such as EPA and DHA, which promote fish health and improve flesh nutritive value for human consumption [1, 2]. However, the increasing demand for FO in the aquafeed industry has put significant pressure on fishery resources and marine ecosystem biology. Consequently, there is growing interest in finding alternative lipid sources to replace FO in aquafeeds, and vegetable oils have emerged as prospective candidates due to their wide range of sources and affordability [3–6]. Among the commonly available vegetable oils, such as soybean oil (SBO), peanut oil (PNO), SFO, corn oil (CO), and canola oil (CNO), limited research has been conducted on their potential as alternative lipid sources to FO.

While SBO, SFSO, and CNO have been extensively studied in aquatic animals, the outcomes of replacing FO with these oils have been inconsistent across different fish species, experimental conditions, and fish sizes [7–10]. Specifically, there is a lack of related studies on the effects of these oils on *Labeo rohita* (*L*. *rohita*), a freshwater species. Moreover, vegetable oils generally contain high levels of oleic acid (OA, C18:1n9) and linoleic acid (LA, C18:2n6) but lack sufficient amounts of LC-PUFAs [6, 11, 12], which play essential roles in lipid metabolism regulation, antioxidant capacity, and immune function in aquatic animals [13–15]. Therefore, replacing FO with vegetable oils may have potential negative effects on fish health and the value of their flesh for humans. However, comprehensive investigations on these indicators have been scarce [16–18]. Some studies on freshwater species have suggested the possibility of substituting high amounts or even the entire fish oil content with vegetable oil without adversely affecting growth performance [3].

Sunflower oil, one of the world’s four major edible oils along with soybean oil, rapeseed oil, and cottonseed oil, is widely used in daily food preparation due to its high content of PUFAs, ranging from 85% to 95% [18]. It is particularly rich in linoleic acid (18:2n-6), which constitutes approximately 68% to 72% of its total fatty acid content and has been associated with various health benefits, including reduced cardiovascular risks [18, 19]. Sunflower oil also contains natural antioxidants such as α-tocopherol and vitamins A, D, and E, which enhance its oxidative stability [18, 20].

Freshwater fish species possess a natural ability to convert C18 PUFAs into longer-chain highly unsaturated fatty acids (HUFAs) within the same series [3, 21]. This metabolic capability opens up opportunities for the successful replacement of fish oil in the diets of freshwater species with certain vegetable oils, such as sunflower oil, which are notably rich in 18:2n-6 [3, 22]. This suggests that freshwater fish may have a higher likelihood of effectively utilizing vegetable oils as a complete substitute for fish oil.

Rohu (*Labeo rohita*) is a highly valuable tropical freshwater carp that is extensively farmed in the Indian subcontinent and other regions worldwide [23, 24]. It holds significant importance in the aquaculture industry, as carp production contributes over 72% to global freshwater aquaculture production, with *L*. *rohita* accounting for approximately 15% of that [25]. Rohu has gained popularity due to its rapid growth rate, increasing market demand, and ability to thrive in diverse agroclimatic conditions. Among the Indian major carps, rohu comprises 35% of the total output and plays a crucial role in carp polyculture systems [26]. The global production of *L*. *rohita* has witnessed substantial growth, reaching 2 million tonnes (valued at USD 3.4 billion) in 2018, up from 1.1 million tonnes (valued at USD 1.6 billion) in 2010. Consequently, it has emerged as a significant aquaculture species, contributing approximately 3.7% to worldwide aquaculture production in 2018 [27].

Despite its economic importance, there is a lack of research on the replacement of FO with SFO specifically in *L*. *rohita*. This study aims to investigate the effects of partially or completely replacing FO with SFO on various aspects of *L*. *rohita*, including growth, proximate composition, blood biochemistry, antioxidant enzyme activity, stress response, and fatty acid profile. By examining these parameters, the study seeks to provide valuable insights into the feasibility and potential consequences of substituting SFO with FO in the diet of *L*. *rohita*.

## Materials and methods

### The experimental diets

The study involved the formulation of five different experimental diets, each with a crude protein content of 30% and crude lipid content of 7%. These diets were formulated using the various feed ingredients listed in Table 1. To ensure consistency, all the diets were designed to have the same nitrogen, lipid, and caloric content. The necessary ingredients, including local grade fish oil, sunflower oil, and others, were obtained from the local market in Lahore, Pakistan. The ingredients were carefully weighed, ground, and mixed using an electrical mixer (KENWOOD, KM 280). Water was added to form a dough, which was then pelletized using a meat mincer (ANEX, AG 3060). The resulting pellets were air-dried and packed in well-sealed plastic bags. They were stored at -20°C until they were ready to be used in the experiment and subsequent analysis. The five experimental diets, named D1, D2, D3, D4, and D5, were created to gradually replace the fish oil with different levels of sunflower oil (0%, 25%, 50%, 75%, and 100%, respectively), as indicated in Table 1. The fatty acid profile of the diets are given in Table 2.

**Table 1:** Ingredients (%) and proximate composition of experimental feeds.

Ingredients %	D1	D2	D3	D4	D5
Fish meal^a^	15.00	15.00	15.00	15.00	15.00
Soybean meal^b^	25.00	25.00	25.00	25.00	25.00
Sunflower meal^b^	20.00	20.00	20.00	20.00	20.00
Corn gluten^c^	11.00	11.00	11.00	11.00	11.00
Wheat flour^d^	10.00	10.00	10.00	10.00	10.00
Rice polish^b^	10.00	10.00	10.00	10.00	10.00
Fish oil^a^	7.00	5.25	3.50	1.75	0.00
Sunflower oil	0.00	1.75	3.50	5.25	7.00
Vitamin premix^e^	1.00	1.00	1.00	1.00	1.00
Mineral mixture^f^	1.00	1.00	1.00	1.00	1.00
**Proximate composition**					
Crude protein%	30.1	30.0	30.1	30.2	30.3
Crude lipids %	11.1	11.1	11.1	11.1	11.1
Moisture %	11.2	11.3	11.2	11.1	11.2
Crude fiber %	6.6	6.6	6.6	6.6	6.6
Dry matter%	89.1	89.0	89.0	89.1	89.0
Ash%	7.3	7.3	7.3	7.3	7.3

D1: control (100% FO); D2: 25% SFO; D3: 50% SFO; D4: 75% SFO; D5: 100% SFO

^a^Paradise Fish Meal, Karachi, Pakistan

^b^Soybean Meal Sharif Solvent, Multan, Pakistan

^c^Rafhan Custard Pvt. Ltd., Faisalabad, Pakistan.

^d^Family Flour Mill, Pattoki, Pakistan.

^e^Mineral mixture contained the following per kilogram; 23750 mg manganese, 75000 mg zinc, copper 5000 mg, cobalt 2000 mg, iodine 2750 mg, selenium 100 mg, magnesium 200000 mg.

^f^Vitamin premix contained the following per kilogram; 10 mg vitamin B12, 100 mg D-Biotin, 1200 mg folic acid, 4000000 IU vitamin A, 480000 IU vitamin D3, 2400 mg vitamin E, 2400 mg vitamin K3, 4000 mg vitamin B1, 4000 mg Niacin, 4000 vitamin B6, 40000 mg vitamin C, 60000 mg inositol, 10000 mg Cal.D. Pantothenate

**Table 2:** Fatty acid profile (% total fatty acids) of experimental diets.

Fatty acids	D1	D2	D3	D4	D5
C14:0	6.93	5.65	4.14	3.08	1.15
C15:0	0.91	0.70	0.50	0.41	0.26
C18:0	3.70	3.60	3.23	3.08	2.55
C16:0	22.3	20.1	16.91	13.60	10.60
C16:1	6.50	4.62	4.20	2.83	1.74
C18:1	15.4	18.11	23.3	25.1	27.09
C20:1n-9	2.51	3.03	3.22	3.23	3.77
C22:1n-9	3.50	3.37	3.31	3.30	3.12
C18:2n-6 (LA)	7.00	15.12	21.5	31.28	41.44
C18:3n-3(ALA)	2.50	2.20	1.90	1.70	1.50
C20:3n-3	1.12	0.92	0.71	0.53	0.42
C20:5n-3(EPA)	10.80	8.81	6.60	4.10	2.61
C22:6n-3(DHA)	16.91	13.80	10.50	7.81	3.91
ΣSFA^1^	33.80	30.05	24.78	20.16	14.56
ΣMUFA^2^	27.9	29.13	34.02	34.46	35.6
Σn-6PUFA^3^	7.00	15.12	21.50	31.28	41.44
Σn-3PUFA^4^	31.3	25.71	19.70	14.11	8.40
DHA + EPA^5^	27.70	22.60	17.10	11.91	6.50

D1: control (100% FO); D2: 25% SFO; D3: 50% SFO; D4: 75% SFO; D5: 100% SFO

^1^SFA: saturated fatty acids

^2^MUFA: mono‐unsaturated fatty acids

^3^n‐6 PUFA: n‐6 poly‐unsaturated fatty acids

^4^n‐3 PUFA: n‐3 poly‐unsaturated fatty acids

^5^DHA+EPA: C22:6n-3 + C20:5n-3

### Ethics statement, fish rearing, and experimental conditions

The protocols and procedures of this study were approved by the animal use and animal care committee of the University of Veterinary and Animal Sciences, Lahore, Pakistan (No. DR/680, dated 14-11-2022).

The experimental conditions were set up in net cages (HAPAs) situated in earthen fishponds at the Department of Fisheries and Aquaculture, Ravi Campus, Pattoki. These cages were installed outdoors in earthen ponds.

To acclimatize the fish, they were kept in circular tanks for a period of two weeks. During this time, they were fed a controlled diet consisting of 100% FO. A total of 375 experimental fish with an average weight of 18.21 ± 0.22 g were then stocked in 15 small net cages (HAPAs) in triplicate. Each cage had dimensions of 1.5 m wide, 2 m long, and 1.5 m deep and contained 25 fish. The fish were fed twice a day at 8:00–8:30 and 16:00–16:30, with a feed amount equivalent to 3% of their body weight. This feeding regimen was maintained for a duration of 90 days.

Throughout the experiment, the physicochemical parameters of the water, including dissolved oxygen (DO), temperature, and pH, were monitored daily. Digital meters/multimeters (Hanna, Romania) were used to measure and record the values. The recorded parameters were as follows: DO (7.1 ± 0.2 mg/L), temperature (28.5 ± 0.3°C), and pH (7.4 ± 0.1).

### Sample collection

At the conclusion of the trial, the fish were weighed to assess their growth performance. Following this, they were subjected to a 24-hour period of fasting. The fish were then anesthetized using MS-222 (Sigma‒Aldrich) at a concentration of 60 mg/L. Once anesthetized, the fish were euthanized by cerebral percussion and harvested for sample collection. In each net cage of the treatment, five fish were carefully selected and appropriately labeled for subsequent whole-body proximate analysis. Additionally, six fish were dissected to obtain muscle and liver samples, which were analyzed to determine the fatty acid profile. For the assessment of antioxidant enzyme activity, five fish were dissected specifically for liver sample collection. Furthermore, nine fish were chosen for blood collection. Blood samples were drawn from the caudal vein using EDTA vacutainers for hematology analysis, while gel vacutainers were utilized for serology purposes. The gel vacutainers were then subjected to centrifugation at 4000 rpm for 15 minutes at a temperature of 4°C to separate the serum. The separated serum was subsequently stored at -20°C until further analysis.

### Growth parameters and biological indices

Prior to being stocked, the fish were their weighed to determine initial weight. At the conclusion of the feeding trial, several growth parameters were calculated using the formulas established by Hopkins [28]. These parameters included FW (final weight), WG (weight gain), WG% (percentage weight gain), FCR (feed conversion ratio), and SGR (specific growth rate). To further analyze the fish, the liver and intestine were dissected from each net cage (hapa) and weighed individually. This allowed for the calculation of HSI (Hepatosomatic index) and VSI (Viscerosomatic index) using the respective formulas for these conformational metrics.


Weightgain=Finalbodyweight(g)−Initialbodyweight(g)

Weightgain%=(Finalweight−initialweight)/(Initialweight)×100

Feed efficiency (FE) = weight gain (g, wet weight)/feed consumed (g, dry weight)

Feed intake (FI, %/day) = 100×dry matter intake×2/[(initial weight +final weight) × rearing days]

FeedConversionratio=FCR=(Totaldryfeedintake(g)/(Wetweightgain(g)

Specificgrowthrate=In(Finalbodyweight−initialbodyweight)/(no.ofdays)×100

Survival rate (%) = 100 × (final fish number/initial fish number).


Hepatosomaticindex(HSI%)=100×liverwetweight(g)/finalbodyweight(g)

Viscerosomaticindex(VSI%)=100×viscerawetweight(g)/finalbodyweight(g)

### Proximate analysis

The formulated feed and fish samples were subjected to analysis following the procedure outlined in AOAC [29]. Several parameters, including moisture, dry matter, ash, crude protein, and crude fat, were determined. To assess moisture content, the samples were dried in an oven (Wise Ven) at a temperature of 105°C for a duration of 12 hours until a constant weight was achieved. The crude protein content was determined using the Kjeldahl method (Kjeltec^TM^ 8100). The crude fat was extracted using Soxhlet extraction (Behro Test 901745) with petroleum ether as the solvent. Ash content was determined by subjecting the samples to a muffle furnace (Vulcan D-550) at a temperature of 600°C for a period of 6 hours.

### Fatty acid analysis

The extraction of lipids from various samples, including fish oil, sunflower oil, feed, fish liver, and muscle, followed the method described by Folch et al. [30]. This involved homogenizing the samples in a mixture of chloroform and methanol (2:1, v/v). To obtain fatty acid methyl esters (FAMEs), gas chromatography (Agilent 6890 N, Santa Clara, USA) was employed based on the methodology outlined by Arslan et al. [31]. Through this process, the fatty acids were converted into their methyl ester forms. To identify the fatty acids, their retention periods were compared to those of reference fatty acids (Supelco 37 Component FAME Mix; Sigma‒Aldrich, Germany).

### Hematology

Blood samples were collected to assess parameters such as RBC, Hb levels, and Hct using an automated hematological analyzer (Celltac α, MEK-6550 Ltd., Japan). All indices and values conformed to standard clinical assessment procedures.

### Blood biochemistry

Serum samples were subjected to analysis using a state-of-the-art automatic biochemical analyzer (Hitachi 7600–110 Ltd., Japan). This advanced instrument allowed for the measurement of various parameters, including TP, ALB, GLO, and the activities of ALT and AST hepatic-associated enzymes.

### Lipid profile

The lipid profile, which includes CHOL, TG, HDL, and LDL, was determined using an automated analyzer (Hitachi 7600–110 Ltd., Japan).

### Stress response

To estimate plasma cortisol levels, an enzyme-linked immunosorbent assay (ELISA) kit (Cat# DKO001; Diametra) was employed.

For the measurement of serum glucose levels, the biuret technique was utilized using a commercial kit purchased from Pars Azmun, based in Tehran, Iran.

### Antioxidants

Liver samples were mixed with ice-cold normal saline. These mixtures were then homogenized on ice for a duration of 10 minutes at 825 g and 4°C. Following homogenization, the samples were centrifuged. In serum and liver, the activity of SOD was determined using the method described by Giannopolitis and Ries [32]. The activity of MDA was assessed according to the procedure outlined by Gatta et al. [33], while the activity of GSH-Px was measured based on the method developed by Civello et al. [34]. CAT activity was measured following the protocol established by Chance and Maehly [35].

### Statistical analysis

The collected data were tested to assess the normality of distribution and the homogeneity of variances. Subsequently, statistical analysis was conducted using SPSS (version 20) for Windows. One-way analysis of variance (ANOVA) was employed to analyze the data, followed by the application of Duncan’s multiple range test (DMRT) for mean comparisons, as described by Steel and Torrie [36]. The results are presented as the mean ± standard error (SE) values. Statistical significance was considered at a threshold of P<0.05.

## Results

### Growth, survival, feed utilization, and biological indices

In the present study, growth parameters are shown in Table 3. The initial IBW was kept nonsignificant in all diet and control groups by careful grading of fish. At the end of the trial, growth performance, survival, feed utilization, and biological indices showed nonsignificant (P>0.05) differences in all diet and control groups by the replacement of FO with SFO in the fish diet.

**Table 3:** Effect of FO replacement with SFO on growth, survival, feed utilization, and biological indices in *L*. *rohita*.

Parameters	D1	D2	D3	D4	D5	P value
^a^IBW (g)	18.2±0.1	18.3±0.3	18.2±0.3	18.3±0.3	18.1±0.1	0.987
^b^FBW (g)	64.1±0.9	63.9±0.6	64.3±0.4	63.1±0.7	62.9±0.2	0.186
^c^WG (g)	45.9±0.5	45.6±0.1	46.1±0.7	44.8±0.9	44.8±0.3	0.400
^d^WG%	251.8±3.2	249.9±3.4	253.1±8.6	245.5±8.6	247.7±2.2	0.893
^e^SGR (%/day)	1.39±0.01	1.39±0.01	1.40±0.02	1.37±0.02	1.38±0.00	0.889
^f^FCR	1.31±0.01	1.35±0.02	1.34±0.03	1.39±0.04	1.37±0.01	0.515
^g^SR (%)	100±0	100±0	100±0	100±0	100±0	-
^h^FI (%/day)	0.7±0.0	0.7±0.0	0.7±0.0	0.8±0.0	0.8±0.0	0.515
^i^FE	0.7±0.0	0.7±0.0	0.7±0.0	0.7±0.0	0.7±0.0	0.524
^j^HSI (%)	1.6±0.0	1.6±0.0	1.6±0.0	1.6±0.0	1.6±0.0	0.602
^k^VSI (%)	10.5±0.1	10.6±0.1	10.5±0.0	10.5±0.0	10.5±0.0	0.790

D1: control (100% FO); D2: 25% SFO; D3: 50% SFO; D4: 75% SFO; D5: 100% SFO

*Superscripts on different means ± SEMs within rows differ significantly at P<0.05

^a^IBW: initial body weight

^b^FBW: final body weight

^c^WG: weight gain

^d^WG%: weight gain percentage

^e^SGR: specific growth rate

^f^FCR: feed conversion ratio

^g^SR: survival rate

^h^FI: feed intake

^i^FE: feed efficiency

^j^HIS: hepatosomatic index

^k^VSI: viscerosomatic index

### Whole body proximate composition

Proximate analysis of the whole body is shown in Table 4. The protein, lipid, ash, moisture and dry matter contents of the fish whole body showed nonsignificant differences (P>0.05), and dietary oil replacement showed the same pattern of results.

**Table 4:** Effect of FO replacement with SFO on the whole body proximate composition (% wet basis) in *L*. *rohita*.

Parameters	D1	D2	D3	D4	D5	P value
Crude protein %	17.4±0.2	17.2±0.2	17.5±0.3	17.1±0.1	17.3±0.1	0.483
Crude lipid %	6.5±0.1	6.5±0.3	7.1±0.2	6.7±0.0	6.8±0.1	0.264
Ash %	3.1±0.2	3.0±0.2	3.0±0.1	3.2±0.1	3.1±0.2	0.798
Moisture %	71.5±0.2	72.0±0.3	72.2±0.6	72.8±0.4	72.1±0.2	0.285
Dry matter %	28.5±0.2	28.0±0.3	27.8±0.6	27.2±0.4	27.9±0.2	0.285

D1: control (100% FO); D2: 25% SFO; D3: 50% SFO; D4: 75% SFO; D5: 100% SFO

*Superscripts on different means ± SEMs within rows differ significantly at P<0.05

### Fatty acid profile of muscle and liver

The effect of dietary SFO substitution with FO in muscle and liver is given in Tables 5 and 6. The SFA composition in muscle and liver decreased (P<0.05) as the substitution level of SFO increased in the diet. The MUFA composition increased (P<0.05) in muscle and liver with increasing SFO levels in the fish diet. The n-6 PUFA composition was also enhanced (P<0.05) by SFO dietary inclusion. The C18:3n‐3 (ALA) composition decreased (P<0.05) in both muscle and liver. Additionally, the C20:5n‐3 (EPA) and C22:6n‐3 (DHA) composition decreased (P<0.05). C18:1n‐9c (Oleic) fatty acids also increased with SFO elevation in diets for both the liver and muscle of *L*. *rohita*.

**Table 5:** Effect of FO replacement with SFO on fatty acid profile (% of total detected) of muscle in *L*. *rohita*.

Fatty acids	D1	D2	D3	D4	D5	P value
C14:0	3.62±0.07^d^	3.50±0.01^d^	2.98±0.00^c^	1.86±0.00^b^	1.52±0.01^a^	0.000
C16:0	19.11±0.02^e^	17.97±0.02^d^	16.95±0.01^c^	15.55±0.01^b^	13.52±0.03^a^	0.000
C18:0	4.11±0.02^d^	3.80±0.01^c^	3.73±0.01^bc^	3.65±0.03^ab^	3.58±0.00^a^	0.000
C24:0	1.04±0.02^ab^	1.09±0.01^b^	1.03±0.02^a^	1.01±0.00^a^	1.04±0.00^ab^	0.075
∑SFA^1^	27.88±0.04^e^	26.37±0.06^d^	24.70±0.05^c^	22.08±0.02^b^	19.68±0.02^a^	0.000
C16:1	3.97±0.01^e^	3.76±0.03^d^	2.98±0.01^c^	2.58±0.02^b^	2.43±0.01^a^	0.000
C18:1n‐9c	23.40±0.01^a^	26.27±0.01^b^	28.96±0.00^c^	30.95±0.01^d^	32.43±0.14^e^	0.000
C20:1	2.59±0.03^b^	2.37±0.00^a^	2.37±0.01^a^	2.37±0.04^a^	2.59±0.06^b^	0.11
C22:1n‐9	0.63±0.00^b^	0.61±0.08^ab^	0.54±0.01^ab^	0.47±0.01^a^	0.48±0.00^a^	0.90
∑MUFA^2^	30.60±0.04^a^	33.02±0.06^b^	34.86±0.04^c^	36.38±0.04^d^	37.94±0.07^e^	0.000
C18:2n‐6c	8.48±0.01^a^	14.90±0.01^b^	17.22±0.01^c^	22.79±0.01^d^	25.67±0.08^e^	0.000
C20:4n‐6	0.88±0.01^c^	0.67±0.01^a^	0.78±0.01^b^	0.67±0.01^a^	0.63±0.01^a^	0.000
∑n‐6PUFA^3^	9.36±0.02^a^	15.57±0.00^b^	18.00±0.00^c^	23.46±0.02^d^	26.30±0.06^e^	0.000
C18:3n‐3(ALA)	4.72±0.01^d^	4.21±0.00^c^	4,22±0.01^c^	4.10±0.01^b^	3.77±0.01^a^	0.000
C20:5n‐3(EPA)	5.26±0.01^e^	3.85±0.00^d^	3.25±0.02^c^	2.71±0.00^b^	2.39±0.00^a^	0.000
C22:6n‐3(DHA)	22.17±0.04^e^	16.97±0.00^d^	14.96±0.01^c^	11.26±0.02^b^	9.90±0.00^a^	0.000
∑n‐3PUFA^4^	32.15±0.02^e^	25.03±0.00^d^	22.43±0.00^c^	18.07±0.01^b^	16.07±0.01^a^	0.000
n-3/n-6	3.43±0.01^e^	1.60±0.00^d^	1.24±0.00^c^	0.77±0.00^b^	0.61±0.00^a^	0.000
n-6/n-3	0.29±0.00^a^	0.62±0.00^b^	0.80±0.00^c^	1.29±0.00^d^	1.63±0.00^e^	0.000
PUFA/SFA	1.48±0.00^a^	1.54±0.00^b^	1.63±0.00^c^	1.88±0.00^d^	2.15±0.00^e^	0.000

D1: control (100% FO); D2: 25% SFO; D3: 50% SFO; D4: 75% SFO; D5: 100% SFO

*Superscripts on different means ± SEMs within rows differ significantly at P<0.05

^1^SFA: saturated fatty acids

^2^MUFA: mono‐unsaturated fatty acids

^3^n‐6 PUFA: n‐6 poly‐unsaturated fatty acids

^4^n‐3 PUFA: n‐3 poly‐unsaturated fatty acids

**Table 6:** Effect of FO replacement with SFO on fatty acid profile (% of total detected) of liver in *L*. *rohita*.

Fatty acids	D1	D2	D3	D4	D5	P value
C14:0	2.86±0.02^c^	2.43±0.02^b^	2.36±0.01^b^	2.18±0.15^b^	1.68±0.03^a^	0.001
C16:0	30.11±0.10^d^	28.37±0.27^c^	28.12±0.36^c^	25.37±0.04^b^	20.98±0.25^a^	0.000
C17:0	1.24±0.02^d^	1.05±0.02^c^	0.91±0.02^b^	1.24±0.03^d^	0.55±0.02^a^	0.000
C18:0	6.54±0.03^a^	6.34±0.08^a^	7.62±0.02^c^	7.21±0.12^b^	6.44±0.02^a^	0.000
∑SFA^1^	40.76±0.09^e^	38.20±0.19^c^	39.01±0.30^d^	36.01±0.04^b^	29.58±0.25^a^	0.000
C18:1n‐9c	10.20±0.05^a^	11.83±0.23^b^	14.22±0.17^c^	17.31±0.04^d^	19.17±0.04^e^	0.000
C16:1	2.43±0.03^d^	2.13±0.02^c^	1.88±0.02^b^	1.70±0.02^a^	1.67±0.02^a^	0.000
C20:1	1.14±0.01^a^	1.18±0.02^a^	1.30±0.02^b^	1.26±0.03^b^	2.41±0.00^c^	0.000
C22:1n‐9	0.33±0.03^a^	0.65±0.04^b^	1.20±0.02^c^	1.69±0.03^e^	1.53±0.06^d^	0.000
∑MUFA^2^	14.11±0.13^a^	15.80±0.23^b^	18.60±0.23^c^	21.97±0.02^d^	24.79±0.08^e^	0.000
C18:2n‐6c	4.74±0.03^a^	9.48±0.17^b^	12.07±0.02^c^	16.98±0.01^d^	22.41±0.13^e^	0.000
C20:4n‐6	3.54±0.01^e^	2.85±0.04^d^	2.56±0.00^c^	2.15±0.04^b^	1.88±0.01^a^	0.000
∑n‐6PUFA^3^	8.28±0.02^a^	12.33±0.13^b^	14.63±0.02^c^	19.13±0.04^d^	24.29±0.12^e^	0.000
C18:3n‐3(ALA)	3.54±0.01^e^	3.11±0.01^d^	2.91±0.02^c^	2.46±0.02^b^	2.21±0.00^a^	0.000
C20:5n‐3(EPA)	4.01±0.06^c^	3.94±0.03^c^	3.23±0.01^b^	2.31±0.07^a^	2.31±0.02^a^	0.000
C22:6n‐3(DHA)	29.29±0.14^e^	26.61±0.07^d^	21.61±0.06^c^	18.11±0.02^b^	16.82±0.03^a^	0.000
∑n‐3PUFA^4^	36.84±0.06^e^	33.66±0.09^d^	27.75±0.09^c^	22.89±0.02^b^	21.34±0.05^a^	0.000
n-3/n-6	4.44±0.02^e^	2.72±0.03^d^	1.89±0.00^c^	1.19±0.00^b^	0.87±0.00^a^	0.000
n-6/n-3	0.22±0.00^a^	0.36±0.00^b^	0.52±0.00^c^	0.83±0.00^d^	1.13±0.00^e^	0.000
PUFA/SFA	1.10±0.00^a^	1.20±0.00^c^	1.08±0.01^a^	1.16±0.00^b^	1.54±0.01^d^	0.000

D1: control (100% FO); D2: 25% SFO; D3: 50% SFO; D4: 75% SFO; D5: 100% SFO

*Superscripts on different means ± SEMs within rows differ significantly at P<0.05

^1^SFA: saturated fatty acids

^2^MUFA: mono‐unsaturated fatty acids

^3^n‐6 PUFA: n‐6 poly‐unsaturated fatty acids

^4^n‐3 PUFA: n‐3 poly‐unsaturated fatty acids

### Hematological and biochemical parameters

Hematology and blood biochemical profiles are presented in Table 7. Blood hematological parameters, such as RBC, Hb, and Hct, showed nonsignificant (P>0.05) changes in all diet and control groups after the replacement of FO with SFO in the fish diets. Blood biochemical parameters such as TP, ALT, and AST also showed nonsignificant (P>0.05) differences among all groups, and dietary oil replacement had no effect on these parameters, while the TP level was significantly (P<0.05) higher in the 100% SFO substitution group than in the other groups and the control group. ALB and GLO showed significant (P<0.05) changes among all dietary and control groups. The ALB level was higher at 100% SFO substitution in the diet compared to 75% SFO substitution and others, and at 0%, 25%, and 50%, the ALB level was nonsignificant (P>0.05) among dietary groups.

**Table 7:** Effect of FO replacement with SFO on hematology and serum biochemistry in *L*. *rohita*.

Parameters	D1	D2	D3	D4	D5	P value
**Hematology**						
^1^RBC (*106 ul-1)	2.88±0.01	2.84±0.06	2.78±0.05	2.83±0.14	2.90±0.02	0.831
^2^Hb (g/l)	8.80±0.20	8.45±0.65	8.35±0.40	8.30±0.10	9.00±0.10	0.635
^3^Hct (%)	31.50±0.20	32.75±0.65	30.30±0.40	30.00±0.10	30.35±1.45	0.183
**Serum Biochemistry**						
^4^TP (g/dl)	2.80±0.10^a^	3.01±0.10^a^	3.19±0.34^ab^	3.60±0.28^ab^	3.90±0.07^c^	0.067
^5^ALB (g/dl)	1.94±0.04^a^	2.00±0.01^a^	2.16±0.05^a^	2.48±0.16^b^	2.92±0.05^c^	0.002
^6^GLO (g/dl)	2.85±0.05^d^	2.71±0.00^c^	2.22±0.01^b^	2.10±0.02^b^	1.92±0.05^a^	0.000
^7^ALT (U/L)	15.50±2.50	15.50±0.50	16.50±0.50	16.35±0.55	16.20±0.60	0.953
^8^AST (U/L)	212.50±0.50	214.50±1.50	215.50±6.50	210.50±0.50	218.00±1.00	0.531

D1: control (100% FO); D2: 25% SFO; D3: 50% SFO; D4: 75% SFO; D5: 100% SFO

*Superscripts on different means ± SEMs within rows differ significantly at P<0.05

^1^RBC: Red blood cells

^2^Hb: Hemoglobin

^3^Hct: Hematocrit

^4^TP: Total protein

^5^ALB: Albumin

^6^GLO: Globulin

^7^ALT: Alanine transaminase

^8^AST: aspartate transaminase.

### Lipid profile and stress biomarkers

The serum lipid profile and stress biomarkers are presented in Table 8. Lipid parameters such as CHOL, TG, and HDL were not significantly (P>0.05) changed in all diet and control groups with respect to dietary oil replacement in the fish diet. LDL levels were significantly changed and increased at 50% SFO, 75% SFO, and 100% SFO substitution in the fish diet compared to the 25% SFO and control groups. Stress biomarkers such as cortisol and glucose levels were found to be similar (P>0.05), and dietary oil replacement showed no effect on these levels.

**Table 8:** Effect of FO replacement with SFO on serum lipid profile and plasma cortisol levels in *L*. *rohita*.

Parameters	D1	D2	D3	D4	D5	P value
**Lipid Profile**						
^1^CHOL (g/dL)	111.00±4.00	112.50±1.50	112.50±0.50	116.00±1.00	118.50±2.50	0.261
^2^TG (g/dL)	153.50±1.50	153.00±3.00	160.00±1.00	160.00±2.00	161.00±4.00	0.185
^3^HDL (g/dL)	49.50±0.50	45.00±2.00	49.50±1.50	46.00±1.00	47.00±1.00	0.176
^4^LDL (g/dL)	12.50±0.50^a^	14.50±0.50^a^	17.40±0.40^b^	18.00±0.30^b^	17.9±1.15^b^	0.006
**Stress Biomarkers**						
^5^CORT (nmol/L)	1422.20±67.40	1370.75±27.85	1430.20±2.30	1429.50±7.70	1439.15±3.05	0.629
^6^GLU (g/dL)	57.50±0.50	56.50±2.50	56.00±1.00	58.50±0.50	59.50±0.50	0.388

D1: control (100% FO); D2: 25% SFO; D3: 50% SFO; D4: 75% SFO; D5: 100% SFO

*Superscripts on different means ± SEMs within rows differ significantly at P<0.05

^1^CHOL: cholesterol

^2^TG: triacylglycerol

^3^HDL: high-density lipoprotein

^4^LDL: low-density lipoprotein

^5^CORT: cortisol

^6^GLU: glucose.

### Antioxidant status in liver and serum

Antioxidant enzyme activity in the liver and serum is presented in Table 9. In the liver, antioxidant enzymes such as SOD, CAT, and GSH-Px showed nonsignificant (P>0.05) changes in activity in all diet and control groups with respect to dietary oil replacement in the fish diet. MDA showed significantly (P<0.05) lower activity with increasing dietary oil replacement in the fish diet compared to the control and at the 25% SFO substitution level. Additionally, serum SOD, CAT, and GSH-Px showed nonsignificant (P>0.05) changes in activity in all diet and control groups with respect to dietary oil replacement in the fish diet. MDA showed significantly (P<0.05) lower activity with increasing dietary oil replacement in the fish diet compared to the control and at the 25% SFO substitution level.

**Table 9:** Effect of FO replacement with SFO on antioxidant enzyme activity in the liver and serum in *L*. *rohita*.

Parameters	D1	D2	D3	D4	D5	P value
**Serum**						
^a^SOD (U/mg protein)	83.00±1.15	83.66±2.02	86.00±1.73	86.66±2.40	89.00±2.08	0.256
^b^MDA (nmol/mg protein)	33.76±1.61^c^	30.46±2.08^c^	25.00±1.13^b^	19.96±0.47^a^	17.70±0.56^a^	0.000
^c^GSH-Px (U/mg protein)	261.86±1.72	256.30±4.01	258.50±3.95	265.86±2.36	266.76±3.49	0.177
^d^CAT (U/mg protein)	13.26±0.48	13.06±0.26	12.60±0.20	12.90±0.37	12.33±0.12	0.312
**Liver**						
SOD (U/mL)	68.66±1.20	69.66±2.60	71.00±1.15	72.00±3.05	73.66±1.76	0.514
MDA (nmol/mL)	1.33±0.08^b^	1.33±0.14^b^	1.03±0.03^a^	0.97±0.01^a^	0.91±0.00^a^	0.007
GSH-Px (U/mL)	94.60±1.32	93.36±0.37	93.76±1.47	92.73±3.53	93.00±1.43	0.962
CAT (U/mg protein)	77.33±1.06	76.63±1.64	75.46±1.39	75.73±1.97	75.33±0.83	0.837

D1: control (100% FO); D2: 25% SFO; D3: 50% SFO; D4: 75% SFO; D5: 100% SFO

*Superscripts on different means ± SEMs within rows differ significantly at P<0.05

^a^SOD, superoxide dismutase.

^b^MDA, malondialdehyde.

^c^GSH-Px, glutathione peroxidase.

^d^CAT, catalase.

## Discussion

Due to the rising cost and declining quality as well as scarcity of FO, there has been a surge in research focused on finding alternative vegetable oils (VOs) that can be used in aquaculture diets. Several studies have revealed that VOs can be successfully incorporated into marine fish diets, either as complete or partial substitutes for FO, without negatively impacting the growth performance of the fish [37, 38]. Regarding dietary modifications, growth stands out as the most prevalent and crucial performance parameter according to the National Research Council [39]. In the present study, SFO was gradually substituted in the *L*. *rohita* diet by replacing FO, and it did not impair fish growth performance or biological indices. The findings of the present study indicate that using vegetable oils such as SFO does not negatively impact the growth performance of *L*. *rohita*. This suggests that the vegetable oils examined in this study have the potential to FO in rahu diets. Similar positive outcomes have been observed in previous studies where the substitution of FO with vegetable oil sources resulted in improved growth and diet efficiency in Nile tilapia [40], juvenile Mozambique tilapia [41], and black carp [42]. Further confirmatory findings were reported by El Asely et al. [43], who fed Nile tilapia SFO instead of FO in the diet, which did not impair fish growth. Nguyen et al. [44] also reported similar results when *Cyprinus carpio* was fed SFO by replacing cod liver oil, and a nonsignificant difference in growth performance was recorded. Furthermore, Rahman et al. [45] also reported no change in growth performance by substituting SFO with FO in the diet of Asian sea bass.

Furthermore, the study also assessed the hepato- and viscera somatic indices, which provide insight into the nutritional status of fish by measuring the liver and viscera weights in relation to the whole-body weight. The liver and viscera serve as energy storage organs [43]. In terms of the HSI and VSI, the results showed nonsignificant changes between the experimental groups. This suggests that the vegetable oils did not have a noticeable impact on the liver and intestine size relative to the whole-body weight of the fish or lipid assimilation efficacy. El Asely et al. [43] discovered comparable outcomes when they fed Nile tilapia a diet where FO was replaced with SFO, as it did not negatively impact the HIS and VSI in the fish. Our finding also aligns with the study conducted by Rahman et al. [45] on Asian seabass, where substituting SFO for FO in the diet showed no changes in HIS and VSI. Consistent with our findings, a previous study by Peng et al. [46] also reported that different dietary sources had no effect on VSI.

In our present study, we analyzed the proximate composition of the whole body, including protein, lipid, ash, moisture, and dry matter content, and found no significant changes in any of the experimental dietary groups or the control group when SFO replaced FO in the diet of *L*. *rohita*. These results are consistent with similar investigations conducted by Jin et al. [14] in sea bream, El Asely et al. [43] in Nile tilapia, Rahman et al. [45] in Asian seabass, and Yu et al. [6] in hybrid sturgeon. In these studies, when gradually or fully substituting SFO in the fish diet for FO, no significant alterations were observed in the proximate composition.

Numerous studies conducted on various fish species have consistently shown that the fatty acid composition of fish tissues reflects the fatty acid composition of their diet [6, 7]. This aligns with our present study; when FO was replaced in the diet, we observed significant changes in the fatty acid composition of their muscle and liver. Specifically, there was an increase in the content of oleic acid (OA) or linoleic acid (LA), while the content of EPA and DHA decreased. EPA and DHA are LC-PUFAs that are known to be beneficial for human health. Fish are considered a major source of EPA and DHA in the human diet. Therefore, it is important to maintain the nutritional quality of farmed fish when replacing dietary fish oil, and this has garnered significant interest [6, 47]. Previous studies have explored the use of vegetable oil-based diets followed by a fish oil finishing diet as a potential approach to address this concern [5]. These studies found that such dietary strategies could improve the EPA and DHA concentrations in the fillets of various fish species, including rainbow trout [5], European whitefish [48], Atlantic salmon [49, 50], red hybrid tilapia [37], European sea bass [51], and red seabream [52]. This approach offers promise in overcoming the challenges associated with replacing fish oil in aquaculture feed [6]. More recently, Davies et al. [53] successfully tested an algal oil enrichment strategy for tilapia in the preharvesting stage to significantly invert the high omega-6: omega-3 ratio in muscle fillets in favor of elevated DHA in a previously high plant and oil diet.

Hematological assessment plays a vital role in monitoring the health status of fish [45, 54]. The hematological characteristics of fish can provide valuable insights into dietary regulation, nutrition, stress, and overall health [14, 55]. RBCs act as both mechanical and biochemical barriers against infections, bacteria, and blood parasites [14, 56]. Furthermore, Hb in aquatic animals binds oxygen across a wide range of partial pressures, facilitating oxygen transport to different tissues in response to environmental conditions and metabolic demands [14, 55, 56]. In our study, we investigated the impact of substituting SFO for FO at different levels in the diet of *L*. *rohita*. We found that the levels of RBCs, Hb, and Hct did not exhibit significant changes. These findings are consistent with similar studies conducted by Jin et al. [14] in juvenile black seabream, Rahman et al. [45] in Asian seabass, and Reda et al. [54] in *Oreochromis niloticus*, where different levels of SFO or complete replacement of FO in the diet showed nonsignificant effects on these hematological parameters.

TP levels were significantly higher in the group with 100% SFO inclusion in the fish diet than in the other dietary and control groups. Additionally, ALB increased while GLO decreased significantly with increasing levels of SFO in the fish diet. Similar findings were reported by Reda et al. [54] in *Oreochromis niloticus* when different levels of SFO or complete replacement of FO were incorporated into the diet. Blood serum protein serves as a sensitive biochemical system reflecting the condition of organisms, which can be influenced by various internal and external factors [45, 57]. Factors such as sex, age, spawning, food, light, temperature, osmotic pressure, hibernation hormones, oxygen depletion, and season can affect the demand for total serum protein [45].

ALT and AST are predominantly metabolic enzymes present in hepatocytes. Increased membrane permeability and elevated serum ALT and AST activities are prime biomarker indicators of liver damage [6, 58]. ALT and AST levels are commonly used as serum markers for diagnosing liver injury, as well as for assessing the secretion of liver proteins [43]. In our study, the levels of ALT and AST showed no significant differences among all dietary and control groups when SFO was included in the fish feed. This finding is consistent with the results of El Asely et al. [43] in Nile tilapia and Yu et al. [6] in hybrid sturgeon when comparing SFO and FO diets. Overall, our findings highlight the stability of hematological parameters and liver enzymes in fish fed diets containing SFO, which aligns with previous studies conducted on various fish species showing no adverse effects.

Fish blood serves as a crucial indicator of metabolism, nutritional status, and disease. When fish are affected by external factors or experience physiological or pathological changes, these changes are often reflected in blood parameters. Although blood lipid content represents only a small fraction of the total body lipid content, it plays a vital role in metabolism [38]. Lipids from both exogenous sources, such as absorbed food lipids, and endogenous sources, such as lipids synthesized by the liver or mobilized from adipose tissue, must pass through the blood to be transported to other tissues. Thus, blood lipid levels can provide insights into overall lipid metabolism in fish [38]. In our study, we examined the serum lipid levels, including CHOL, TG, HDL, and LDL, in *L*. *rohita* fed diets with SFO substituted for FO. We found that the serum levels of CHOL, TG, and HDL did not exhibit significant changes, while LDL levels were significantly higher in the SFO-fed group than in the FO-fed group. Similar findings were reported by Jin et al. [14] in juvenile black seabream, where CHOL levels remained the same, and by Yu et al. [6] in hybrid sturgeon, where cholesterol, TG, and HDL levels were comparable, but LDL levels were higher in fish fed SFO than in those fed FO. It is well known that the HDL: LDL ratio is higher in animals and humans receiving EPA and DHA in the diet, as described in the recent systematic review by Wang et al. [59]. Elevated dietary levels of LDL in fish seem to also conform to this mechanism of the blood transport profile of lipoprotein carrier complexes, thereby reducing this ratio. Additionally, the stress biomarkers cortisol and glucose showed similar levels in all dietary and control groups, consistent with the results reported by Yu et al. [6] in hybrid sturgeons.

Assessing oxidative function is a significant approach to evaluating the welfare of aquatic species under stressful conditions [60, 61]. Oxidative stress arises from the imbalance between the generation of reactive oxygen species (ROS) and the protective antioxidant defense system [62, 63]. ROS are produced continuously in living organisms and can be harmful, particularly under toxic conditions [64]. Antioxidant enzymes such as CAT, SOD, and GPx play a crucial role in scavenging or neutralizing free radicals (ROS) that can damage cell membranes when present in excessive amounts [62, 65]. MDA is a metabolite derived from lipid peroxidation, specifically from the oxidation of fatty acids with multiple methylene interrupted double bonds [14, 43, 66, 67]. In our study, substituting SFO for FO in the fish diet did not lead to changes in the activity of SOD, CAT, and GSH-Px in the serum and liver, while MDA activity increased. Similar findings were reported by Jin et al. [14] in juvenile black seabream, where SOD and GSH-Px levels remained unchanged while MDA levels decreased in the liver and serum. Similarly, El Asely et al. [43] observed no changes in SOD and GPx activity in Nile tilapia, Reda et al. [54] reported no alterations in SOD and GPX activity in *Oreochromis niloticus*, and Yu et al. [6] found that in hybrid sturgeon, GSH-Px and SOD were the same when fed by substituting SFO in the fish diet by replacing FO.

## Conclusion

In conclusion, fully replacing fish oil with SFO has shown potential for complete FO substitution without compromising growth and overall health status. Interestingly, *L*. *rohita* exhibited a preference for using SFO as a lipid source, which resulted in improved growth performance and did not affect the serum biochemistry or antioxidant and immune capacity. Furthermore, the fatty acid composition of the *L*. *rohita* muscle and liver closely mirrored the composition of their dietary fatty acids. Our findings suggest that careful selection of alternative lipid sources can have a significant impact on the growth, health, and nutritional quality of *L*. *rohita*. These insights contribute to the development of sustainable aquaculture practices that prioritize the replacement of FO with suitable alternatives, ensuring the well-being of fish populations and providing consumers with healthy and high-quality fish options.
